# PINK1/Parkin-Mediated Mitophagy Participates in High-Altitude Hypoxia Adaptation in Yaks via Energy Metabolism Remodeling

**DOI:** 10.3390/ani16142121

**Published:** 2026-07-08

**Authors:** Zheng-Bo Li, Tian-Shuai Li, Miao-Ran Li, Zhong-Duo Li, Jia-Lin Wang, Guo-Xiu Li, Jian-Shu Lv, Ling-Xia Li, Xiang-Dong Ye, Xiao-Dong Ling

**Affiliations:** 1 College of Agriculture and Animal Husbandry, Qinghai University, Xining 810016, China; ys240906010545@qhu.edu.cn (Z.-B.L.); lts431893827@163.com (T.-S.L.); limiaoran0416@163.com (M.-R.L.); 18238653007@163.com (Z.-D.L.); 18434898840@163.com (J.-L.W.); liguoxiu1122@163.com (G.-X.L.); lvjs153@163.com (J.-S.L.); lilingxia852@126.com (L.-X.L.); 2 Gaomiao Town Animal Husbandry and Veterinary Station, Ledu District Agriculture and Rural Affairs Bureau, Haidong 810799, China; xdongye@126.com

**Keywords:** hypoxia, mitophagy, normoxia, PINK1/Parkin, skeletal muscle satellite cells, yaks

## Abstract

Yaks are an ideal model for investigating mammalian adaptation to high-altitude hypoxia; however, the underlying adaptive mechanisms remain unclear. Therefore, this study aimed to investigate the mechanisms underlying yak adaptation to high altitudes, focusing on the role of the PINK1/Parkin pathway. In particular, we established normoxic and hypoxic models of yak skeletal muscle satellite cells (SMSCs). In addition, we examined the relationship between PINK1/Parkin-mediated mitophagy and the activities of key enzymes involved in mitochondrial energy metabolism. At the tissue level, an increase in altitude significantly decreased p62 expression and upregulated LC3B-II expression. Notably, mitophagy levels and the expression of PINK1 and Parkin in the skeletal muscle, lungs, and myocardial tissues increased with altitude. At the cellular level, hypoxia markedly elevated the expression of PINK1 and Parkin proteins in SMSCs, induced the conversion of LC3-I to LC3-II, decreased p62 accumulation, significantly increased the number of autolysosomes, and enhanced autophagic flux. Hypoxia altered mitochondrial electron transport chain function, resulting in compensatory elevation of key enzyme activities in the mitochondrial respiratory chain. However, 3-methyladenine (3-MA) treatment reversed these hypoxia-induced changes. In conclusion, yak SMSCs sustain mitochondrial functional homeostasis under hypoxia via PINK1/Parkin-mediated mitophagy, effectively managing hypoxic stress.

## Simple Summary

Yaks thrive in the hypoxic environment of the Qinghai–Tibet Plateau by employing distinct adaptive strategies for survival. Extended exposure to low-oxygen conditions can adversely affect mitochondria, the cellular organelles responsible for energy generation. Therefore, a comprehensive understanding of the mechanisms underlying hypoxic adaptation in yaks is essential for elucidating how they endure and sustain normal physiological functions at high altitudes. This study investigates whether the mitochondrial quality-control process, specifically PINK1/Parkin-mediated mitophagy, is implicated in yak adaptation to high-altitude hypoxia. Our research shows that yaks eliminate damaged mitochondria and alter energy-generation pathways to endure low oxygen availability. They use PINK1/Parkin-mediated mitophagy to clear damaged mitochondria, helping maintain energy balance under hypoxic conditions. This process supports their musculature, lungs, and myocardium by regulating key energy enzymes.

## 1. Introduction

The Qinghai–Tibetan Plateau features extreme conditions, such as cold alpine temperatures, low oxygen levels, and intense ultraviolet radiation, all of which significantly threaten the survival and reproduction of land animals [1,2]. Yak (*Bos grunniens*) is a large ruminant endemic to the Qinghai–Tibet Plateau [3]. During evolution, yaks have progressively adapted to this harsh environment, rendering them an exemplary biological model for investigating the mechanisms underlying high-altitude hypoxia adaptation in mammals [4,5,6]. Primary stress caused by hypoxia disrupts energy metabolism and creates an imbalance in oxidative stress due to an inadequate oxygen supply [7,8,9]. Skeletal muscle, which acts as an effector of movement and is a key tissue in energy metabolism, relies heavily on oxygen and ATP supply [10,11]. However, the mechanisms by which yaks regulate metabolic networks under hypoxic stress to maintain energy homeostasis in skeletal muscle satellite cells (SMSCs) and inhibit cell damage caused by excessive reactive oxygen species (ROS) accumulation remain unclear.

Mitochondria are the central hub of energy metabolism in eukaryotic cells and are the principal sites for oxidative phosphorylation and ATP generation, as well as the key loci for oxygen sensing and ROS production [12]. Under normal physiological conditions, ROS can function as secondary messengers and positively regulate cellular proliferation and differentiation [13,14]. In hypoxic environments, dysfunction of the electron transport chain leads to increased electron leakage, resulting in a sudden buildup of ROS [15]. Energy supply is compromised when mitochondria are not swiftly and effectively cleared by quality control processes, ultimately triggering cell apoptosis. Peroxisome proliferator-activated receptor-γ coactivator-1α (PGC-1α) is a pivotal regulator of mitochondrial biogenesis and energy metabolism, and alterations in its expression and activity immediately affect mitochondrial functional homeostasis [16,17]. Mitochondrial respiratory chain complex I (MRCCI), succinate dehydrogenase (SDH), and cytochrome c oxidase subunit 1 (COX-1) are essential components of the oxidative phosphorylation pathway [18,19,20]. Notably, the activities of these enzymes serve as indicators of mitochondrial energy metabolism efficiency. In addition, the activity of citrate synthase (CS), the rate-limiting enzyme of the tricarboxylic acid (TCA) cycle, is positively correlated with mitochondrial content [21]. Overall, these enzymes are central hubs in the mitochondrial energy metabolism network and serve as vital indicators of mitochondrial functions.

Mitophagy plays a vital role in maintaining mitochondrial quality [22,23,24] by identifying, engulfing, and degrading damaged or aging mitochondria via the autophagic–lysosomal pathway, thereby ensuring that mitochondrial function remains balanced [25]. Importantly, the role of PINK1/Parkin-mediated mitophagy in neurodegenerative and cardiovascular diseases has been established [26,27,28]. PINK1-mediated mitophagy plays critical roles in various pathological conditions. In a PC12 cell model of Parkinson’s disease, Xu et al. [29] reported that PINK1-dependent mitophagy serves as an effective protective response under hypoxic conditions. In amyotrophic lateral sclerosis (ALS), which involves mitochondrial dysfunction, Wong et al. [30] observed that PINK1 accumulates on the outer mitochondrial membrane and triggers mitophagy through autophosphorylation to eliminate damaged mitochondria. Additionally, Rehman et al. [31] demonstrated that PINK1/Parkin knockout animals develop an exacerbated pulmonary hypertension phenotype upon hypoxic exposure, further supporting the importance of this pathway in hypoxia-related disorders. Therefore, hypoxia can cause mitochondrial damage, activating PINK1/Parkin-mediated autophagy to preserve mitochondrial health and quality. However, the role of PINK1/Parkin-mediated mitophagy in yak adaptation to high-altitude hypoxia remains unclear. In particular, whether the mitophagy pathway improves mitochondrial function and quality, thereby enabling adaptation to hypoxia, remains unclear.

Therefore, this study aimed to investigate the role of PINK1/Parkin-mediated mitophagy in yak adaptation to high-altitude hypoxia using molecular biology, cell biology, and other research methods. In addition, we explored the mechanisms by which mitophagy optimizes mitochondrial function and quality control to promote hypoxic adaptation, providing novel theoretical insights into the adaptation of yaks to high-altitude hypoxia.

## 2. Materials and Methods

### 2.1. Animals

Three healthy 12-month-old male domestic yaks (mean body weight: 125.3 ± 8.2 kg at 2200 m, 118.7 ± 9.5 kg at 3200 m, and 108.4 ± 7.8 kg at 4200 m) were selected from each altitude in Qinghai Province, China, for this study. Skeletal muscle, myocardium, and lung tissues were collected from yaks during slaughter. All animal experiments were approved by the Animal Welfare and Research Ethics Committee of Qinghai University (Number: SL-2023005). All animal experiments in this study were conducted in accordance with the policies and legal requirements of the European Communities Council Directive 2010/63/EU. Additionally, we follow the guiding principles of the Chinese Legislation on the Use and Care of Laboratory Animals.

### 2.2. Cell Culture

Yak SMSCs isolated in our laboratory in a previous study (Supplementary Materials S1) were subjected to normoxic (20% O_2_) or hypoxic (1% O_2_) conditions for durations of 24 and 48 h. The hypoxic oxygen concentration of 1% O_2_ and exposure durations were selected based on previous studies establishing this level as representative of severe hypoxia for in vitro satellite cell models [32,33,34].

### 2.3. Protein Extraction and Western Blot Analysis

Total protein was extracted from yak tissues and SMSCs using the Minute™ Total Protein Extraction Kit for Animal Cells/Tissues (Invent Biotechnologies, Beijing, China, cat. no.: SD-001/SN-002). Protein concentration was quantified using a BCA protein assay kit (Service Bio, Wuhan, China; G2026). Thereafter, proteins were separated using 4–20% sodium dodecyl sulfate–polyacrylamide gel electrophoresis and transferred onto PVDF membranes. After blocking with the appropriate blocking solution, the membranes were incubated at 4 °C overnight with the following primary antibodies: PINK1 (1:1000 dilution, Bioss, Beijing, China, bs-22173), Parkin (1:1000 dilution, Bioss, Beijing, China, bsm-61396R), p62 (1:5000 dilution, Proteintech, Wuhan, China, 18420-1-AP), LC3B (1:1000 dilution, MBL, Tokyo, Japan, PM036), β-actin (1:5000 dilution, Bioss, Beijing, China, bs-0061R), and GAPDH (1:2000 dilution, Servicebio, Wuhan, China, ZB15004-HRP). Thereafter, the membranes were incubated with goat anti-rabbit IgG H&L, IRDye 800cw conjugated secondary antibody (1:15000 dilution, Bioss, Beijing, China, bs-40295G-IRDye800CW). Antibody reactions were detected using an ultrasensitive ECL chemiluminescence kit (Beyotime, Shanghai, China, P0018FS) and visualized using a ChemiDoc imaging system (Bio-Rad, Hercules, CA, USA, 12003153).

### 2.4. mRFP-GFP-LC3 Transfection

Cells in the normoxic and hypoxic groups were seeded into confocal culture dishes and cultured for 24 h to allow for cell attachment. Thereafter, the cells were transfected with an autophagy dual-labeled adenovirus (Hanbio, Shanghai, China; HBAD-mRFP-GFP-LC3) according to the manufacturer’s instructions.

### 2.5. Transmission Electron Microscopy (TEM)

Briefly, the samples were fixed in 2.5% glutaraldehyde for 4 h, rinsed three times with phosphate buffer, and post-fixed in 1% osmium tetroxide at 4 °C for 2 h. After three rinses in ddH_2_O, the samples were dehydrated through a graded ethanol series, transitioned via propylene oxide, gradually infiltrated with 812 resin, embedded, and polymerized at 60 °C. Thereafter, the embedded blocks were cut into semi-thin sections for localization and ultra-thin sections using an ultramicrotome (Leica UC7, Wetzlar, Germany). Finally, the sections were double-stained with uranyl acetate and lead citrate and examined using transmission electron microscopy (JEM1400, JEOL, Tokyo, Japan).

### 2.6. Enzyme-Linked Immunosorbent Assay (ELISA)

ELISA kits were used to measure the activities of CS (Jiangsu Meimian Industrial Co., Ltd., Yancheng, China, MM-001301), CAT (Jiangsu Meimian Industrial Co., Ltd., Yancheng, China, MM-5046301), SDH (Jiangsu Meimian Industrial Co., Ltd., Yancheng, China, MM-5104601), MRCCI (Jiangsu Meimian Industrial Co., Ltd., Yancheng, China, MM-1019V1), and COX-1 (Jiangsu Meimian Industrial Co., Ltd., Yancheng, China, MM-1236V1) and the levels of PGC-1α (Jiangsu Meimian Industrial Co., Ltd., Yancheng, China, MM-1192V1) in cells in the normoxia, hypoxia, and autophagy inhibition groups, according to the manufacturer’s instructions.

### 2.7. Assessment of Total Antioxidant Capacity (T-AOC)

Briefly, the T-AOC of each group was measured using a T-AOC assay kit (BoxBio, Beijing, China, AKAO012M). Finally, T-AOC was calculated using the following formula:

$$T-\mathrm{AOC}\left(\mu \mathrm{mol}/10^{4}\;cell\right)=\frac{x\times V_{\mathrm{reaction}\_\mathrm{total}}\times V_{\mathrm{sample}\_\mathrm{total}}}{V_{\mathrm{sample}}\times \mathrm{cell}\;\mathrm{number}}=\frac{20\times x}{\mathrm{cell}\;\mathrm{number}}$$

where $V_{sample\_total}$ indicates the total volume of the sample to be tested, $V_{reaction\_total}$ indicates the total volume of the reaction system, and $V_{sample}$ denotes the volume of the test sample added to the reaction system [35]. T-AOC values were calculated according to the kit protocol and expressed in µmol/10^4^ cells.

### 2.8. Data Analysis

Data were statistically analyzed using GraphPad 10.4.2 software. Data are presented as mean ± standard deviation (SD) of values from at least three independent experiments. Prior to analysis, the data were subjected to normality and homogeneity of variance tests. The results confirmed that all data were normally distributed (*p* > 0.05 for all groups) and that variances were homogeneous across treatment groups (*p* > 0.05 for all comparisons), satisfying the assumptions for parametric statistical analyses. Significant differences between groups were determined using one-way analysis of variance (ANOVA), followed by Dunnett’s test for post hoc multiple comparisons. Two-group comparisons of data obtained from in vitro cell experiments were performed using an independent t-test. Statistical significance was set at *p* < 0.05.

## 3. Results

### 3.1. Mitophagy Levels in Oxygen-Sensitive Tissues in Yaks Increased with Increasing Altitude

**Figure 1:**
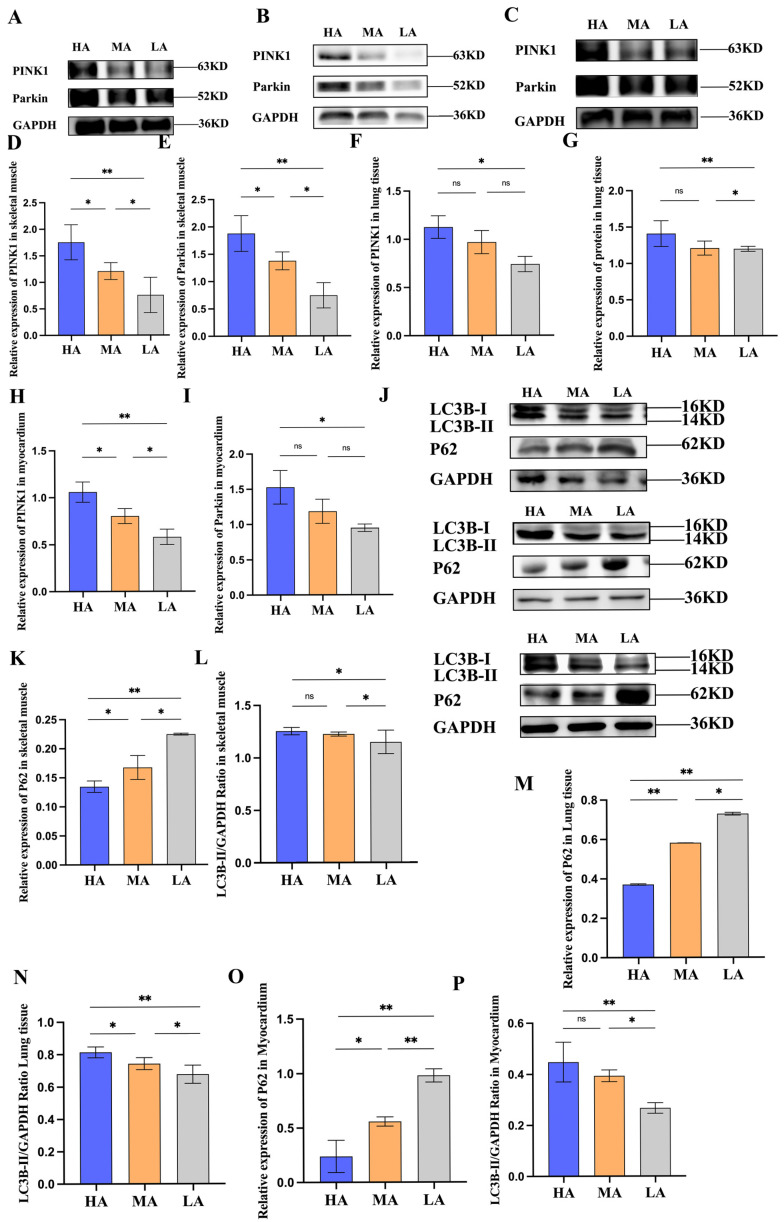
Mitophagy is enhanced in oxygen-sensitive tissues of yaks at higher altitudes. Higher altitudes (HA), Mid-altitude (MA) and low altitude (LA), respectively. After tissue protein extraction, the expression levels of PINK1 and Parkin proteins were assessed by Western blot, and protein quantification was performed using the bicinchoninic acid (BCA) assay with a loading amount of 20 µg. The samples derive from the same experiment. *n* = 3, * *p* < 0.05, ** *p* < 0.01, ns, not significant. (**A**–**C**) Western blot analysis of PINK1 and Parkin protein expression in tissues at different altitudes. (**D**,**E**) Expression of PINK1 and Parkin proteins in skeletal muscle tissue across different altitudes. (**F**,**G**) Expression of PINK1 and Parkin proteins in lung tissues at varying altitudes. (**H**,**I**) Expression of PINK1 and Parkin proteins in myocardial tissues at different altitudes. (**J**) Western blot detection of p62 and LC3B protein expression in tissues at varying altitudes. (**K**,**L**) Expression of p62 and LC3B proteins in lung tissues at varying altitudes. (**M**,**N**) Expression of p62 and LC3B proteins in lung tissues at different altitudes. (**O**,**P**) Expression of p62 and LC3B protein in cardiac tissue at different altitudes.

Western blotting revealed that PINK1 and Parkin expression levels in the skeletal muscle of yak increased with increasing altitude (Figure 1A–C), with significant differences (*p* = 0.004) observed between the high- and low-altitude groups (Figure 1D,E). Similarly, the expression of both proteins in lung tissue exhibited altitude-dependent upregulation, with significant differences (*p* = 0.027) in PINK1 and Parkin expression observed between the high- and low-altitude groups (Figure 1F,G). In addition, the levels of PINK1 and Parkin proteins in myocardial tissue increased with altitude, with significant differences (*p* = 0.017) in PINK1 and Parkin expression between the high- and low-altitude groups (Figure 1H,I).

Further analysis of autophagy markers revealed that p62 levels in skeletal muscle, lung, and heart tissues were significantly lower (*p* = 0.005) in the high-altitude group than in the low-altitude group (Figure 1J,K,M,O). Similarly, the LC3B-II/GAPDH ratio increased in all tissues with increasing altitude (*p* = 0.009; Figure 1L,N,P), indicating a marked increase in LC3B-II expression. Collectively, these results suggest that mitophagy levels in yak tissues increase with altitude, possibly by activating the PINK1/Parkin signaling pathway.

### 3.2. Hypoxia Upregulates Autophagy in Yak SMSCs

**Figure 2:**
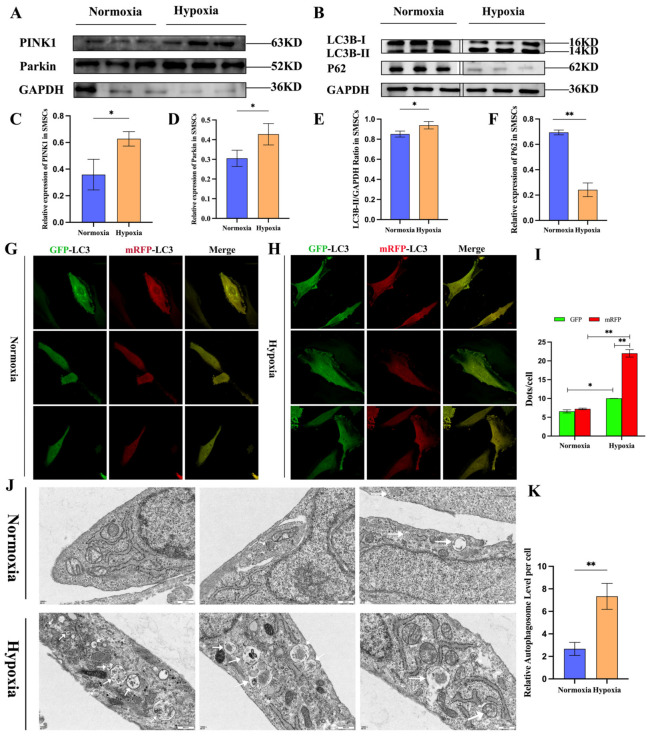
Hypoxia treatment promotes elevated autophagy levels in yak SMSCs. Proteins were extracted from the normoxia (20% O_2_) and hypoxia (1% O_2_) groups after 24 h of cell culture, and Western blot analysis was performed to assess the expression levels of Pink1, Parkin, p62, and LC3B. Protein quantification was performed using the BCA assay, with a loading amount of 20 µg. The samples derive from the same experiment. When transducing cells with mRFP-GFP-LC3, the two groups of cells were separately cultured in confocal dishes for 24 h using the half-volume infection method at an MOI of 100. $Amount\;of\;virus\;added\;(µL/per\;well)=\frac{MOI\times cell\;number}{virus\;titer\;(PFU/mL)}\times 1000$. After 4 h of infection, the culture medium was supplemented to the complete volume. At 24 h post-infection, a second round of viral suspension was added for re-infection. Fluorescence was examined by confocal microscopy (60×) (scale bar = 10 µm). After microscopic imaging, red and green fluorescent signals were merged: yellow puncta indicated autophagosomes, while red puncta indicated autolysosomes. Autophagic flux intensity was assessed by counting puncta of different colors. Ten images were randomly selected from each group for statistical analysis of fluorescence intensity. Cell morphology and ultrastructure were observed by TEM at 10,000× magnification (scale bar = 500 nm). The white arrow indicates an autophagosome. The same ten images were used for statistical analysis. *n* = 3; * *p* < 0.05; ** *p* < 0.01. (**A**,**B**) Expression of PINK, Parkin, LC3B, and p62 proteins in SMSCs under normoxic and hypoxic conditions. (**C**–**F**) Grayscale analysis of Western blot bands. (**G**) Confocal microscopy of mRFP-GFP-LC3 adenoviral fluorescence under normoxia. (**H**) Confocal microscopy of mRFP-GFP-LC3 adenoviral fluorescence under hypoxia. (**I**) Statistics of fluorescence intensity. (**J**) TEM observation of cell morphology. (**K**) Electron-microscopic quantification of autophagosomes and autolysosomes.

Western blot analysis of yak SMSCs exposed to normoxia (20% O_2_) and hypoxia (1% O_2_) revealed that PINK1, Parkin, and LC3-II protein levels and LC3-II/LC3-I ratio were significantly upregulated (*p* = 0.022) in the hypoxic group compared with those in the normoxic group (Figure 2A–E). In contrast, p62 expression was significantly downregulated (*p* = 0.047) in the hypoxic group (Figure 2F), indicating that hypoxic environments activate the PINK1/Parkin pathway and promote autophagy.

In addition, hypoxia-induced autophagy was verified using mRFP-GFP-LC3 dual-fluorescence tracing. In the normoxic group, the cytoplasm predominantly appeared yellow with sporadic red puncta (Figure 2G), indicating that the cells were at the basal level of autophagy. In contrast, the hypoxic group displayed dense fluorescent puncta with significantly increased (*p* = 0.008) yellow and red puncta compared with the normoxic group (Figure 2H,I). TEM confirmed that the number of autophagosomes and autolysosomes was significantly higher (*p* = 0.005) in the hypoxic group than in the normoxic group (Figure 2J,K). Overall, these data indicate that hypoxic conditions induce autophagy and promote autophagic flux in yak SMSCs.

### 3.3. Mitophagy Drives Remodeling of Mitochondrial Energy Metabolism Under Hypoxia

**Figure 3:**
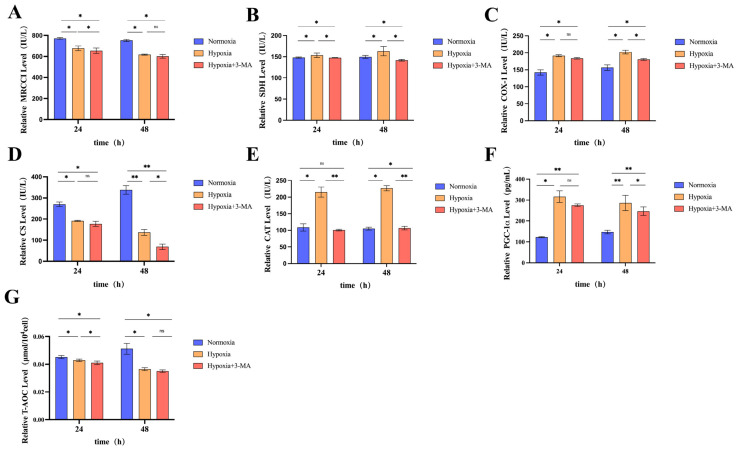
Mitophagy drives the remodeling of mitochondrial energy metabolism during hypoxia. When the cell density in each group reached approximately $10^{6}$ cells/mL, the cells were collected, washed, disrupted by ultrasonication, centrifuged, and the supernatant was collected. After sample loading, use blank wells for zero calibration, and sequentially measure the absorbance (OD) of each well at 450 nm. The sample concentration is determined from the standard curve and then multiplied by the dilution factor to calculate the actual concentration. The cell count for T-AOC detection was $10^{4}$, the absorbance was measured at 593 nm, and the sample concentration (µmol/mL) was calculated. 3-MA was employed to inhibit autophagy at a working concentration of 5 mM, prepared by dissolution in DMSO. *n* = 3, * *p* < 0.05, ** *p* < 0.01, ns, not significant. (**A**–**F**) MRCCI, SDH, COX-1, CS, and CAT activity assays under normoxia, hypoxia, and hypoxia with autophagy inhibition. (**G**) T-AOC was assessed under normoxia, hypoxia, and hypoxic conditions with autophagy inhibition.

To investigate the effects of a hypoxic environment on mitochondrial function in yak SMSCs, we examined changes in the activities of key mitochondrial respiratory chain enzymes (MRCCI, SDH, COX-1, and CS), PGC-1α content, and T-AOC 24 and 48 h after hypoxia treatment. Importantly, hypoxia for 24 h significantly decreased (*p* = 0.028) MRCCI and CS activity and the T-AOC in yak SMSCs compared with normoxic conditions (Figure 3A,D,G). In contrast, SDH, COX-1, and CAT activities and PGC-1α levels in yak SMSCs exhibited a significant compensatory increase after 24 h of hypoxia (*p* = 0.045; Figure 3B,C,E,F). In addition, hypoxia treatment for 48 h significantly decreased MRCCI activity and T-AOC, further increased SDH, COX-1, and CAT activities, significantly downregulated CS activity, and significantly upregulated PGC-1α levels (*p* = 0.027; Figure 3A–G). Collectively, these results indicate that yak SMSCs actively respond to oxidative stress and preserve mitochondrial function under hypoxic stress by regulating the activity of essential enzymes in the mitochondrial respiratory chain.

To investigate the role of hypoxia-induced autophagy, SMSCs were treated with the autophagy inhibitor 3-methyladenine (3-MA). Notably, 3-MA-induced autophagy inhibition significantly decreased MRCCI and SDH activities, T-AOC, and CAT activity in the normoxic group compared with the hypoxia group at 24 h, although no significant differences (*p* = 0.073) were observed in COX-1 and CS activities or PGC-1α content (Figure 3A–G). Although no significant differences (*p* = 0.087) in T-AOC and MRCCI activity were observed between the normoxic and hypoxic groups at 48 h after autophagy inhibition (Figure 3A,G), SDH, COX-1, and CS activities, PGC-1α levels, and CAT activity were significantly lower (*p* = 0.016) in the normoxic group than in the hypoxic group (Figure 3B–F). Overall, these results indicate that hypoxic stress impairs mitochondrial electron transport chain function in yak SMSCs, and autophagy may play a crucial role in preserving mitochondrial function and quality in these cells.

### 3.4. Mitophagy in Yak SMSCs Is Linked to the PINK1/Parkin Pathway

**Figure 4:**
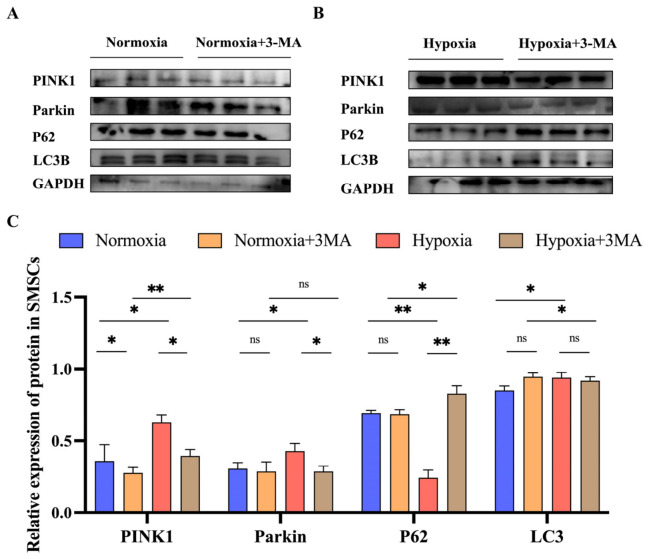
Mitochondrial autophagy in yak SMSCs is coupled to the PINK1/Parkin pathway. The normoxia group (20% O_2_), hypoxia group (1% O_2_), and hypoxia combined with 3-MA group (1% O_2_) were cultured for 24 h, after which proteins were extracted. Autophagy was inhibited with 3-MA at a working concentration of 5 mM. *n* = 3, * *p* < 0.05, ** *p* < 0.01, ns, not significant. Western blot analysis was performed to assess the protein expression levels of Pink1, Parkin, p62, and LC3B. Protein quantification was performed using the BCA assay, with a loading amount of 20 µg. (**A**) Protein expression of PINK1, Parkin, p62, and LC3B under normoxia and normoxia with autophagy inhibition. (**B**) Protein expression of PINK1, Parkin, p62, and LC3B under hypoxia and hypoxia with autophagy inhibition. (**C**) Quantitative analysis of related proteins.

Changes in the expression levels of autophagy-related proteins in SMSCs treated with 3-MA under normoxic and hypoxic conditions were examined using Western blotting (Figure 4A,B). Autophagy inhibition significantly decreased the protein levels of PINK1 and LC3B-II under normoxic conditions (*p* = 0.023), although no significant differences (*p* = 0.052) in Parkin and p62 levels were observed before and after autophagy inhibition (Figure 4C). Under hypoxic conditions, autophagy inhibition significantly downregulated PINK1 and Parkin levels (*p* = 0.041) and significantly upregulated p62 expression (*p* = 0.007); however, no significant differences (*p* = 0.061) were observed in LC3B expression (Figure 4C). Collectively, these results demonstrate that mitophagy is associated with the PINK1 and Parkin pathways in yak SMSCs.

## 4. Discussion

**Figure 5:**
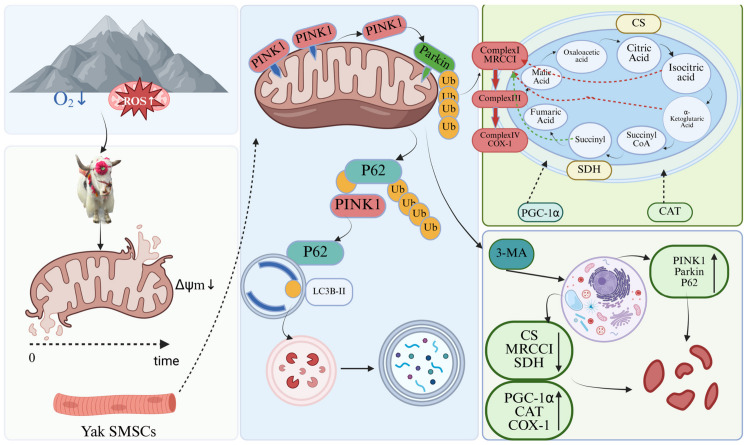
Mechanism of PINK1/Parkin-mediated mitophagy and remodeling of energy metabolism. Created in BioRender. Lee, Z. (2026) https://BioRender.com/f2p25ni (accessed on 15 April 2026). Under high-altitude hypoxia, the accumulation of reactive oxygen species (ROS) triggers mitochondrial depolarization. Damaged mitochondria trigger the PINK1/Parkin pathway, initiating a process known as mitophagy. At this stage, cells reduce metabolic burden and electron leakage by downregulating the entry-point enzymes (CS/MRCCI/SDH) while concurrently upregulating PGC-1α, COX-1, and CAT. This coordinated regulation facilitates efficient biosynthesis alongside antioxidant defense mechanisms, thereby ultimately enhancing hypoxia adaptation. Inhibition of the autophagy pathway causes a significant buildup of damaged mitochondria, leading to a sharp increase in ROS levels. This surge disrupts the stability of the respiratory chain enzymes, further impairing cellular biosynthesis and protective functions, and ultimately causing widespread metabolic dysregulation.

In this study, we elucidated the relationship between mitochondrial quality control and autophagy in yaks and validated the role of PINK1/Parkin-mediated mitophagy in hypoxic adaptation in yaks (Figure 5).

High-altitude hypoxic environments are critical stressors that impact animal survival and health [36,37,38]. Previous studies have established that hypoxia induces autophagy [39,40]. The PINK1/Parkin pathway is a canonical mitophagy regulatory pathway in mammals that senses a decline in the mitochondrial membrane potential, recruits Parkin to damaged mitochondria, and initiates autophagosome engulfment and lysosomal degradation [29,41,42]. Alterations in LC3B and p62 expression are key indicators of changes in autophagosome formation, with autophagic dysfunction inducing p62 accumulation [43,44]. The conversion of LC3 from its unconjugated form (LC3-I) to its phosphatidylethanolamine (PE)-conjugated form (LC3-II) is critical for autophagosome formation [45]. Our study revealed that an increase in altitude (hypoxic environment) significantly upregulated LC3B-II expression in the skeletal muscle, lung tissue, and cardiac muscle of yaks and markedly downregulated p62 expression, indicating increased autophagy. It is well established that hypoxia induces autophagy through HIF-1α-mediated transcriptional upregulation of BNIP3 and through AMPK-dependent mTOR inhibition [46]. Importantly, the expression of PINK1 and Parkin in skeletal muscle, myocardium, and lung tissues increased with altitude, suggesting that the elevation of autophagy levels in yak tissues with increasing altitude may be associated with PINK1/Parkin-mediated mitophagy. Chi et al. [47] reported similar findings in the nervous system of rats with EHS. In a hypoxia-treated yak SMSC model, autophagy levels increased significantly, and autophagy flux remained unimpeded, consistent with the findings of Lv et al. [48] in B-NHL cells. Notably, the parallel upregulation of LC3B II and downregulation of p62 in these tissues mirror the autophagic responses to acute hypoxia in cultured yak SMSCs, suggesting that the same quality-control pathway is activated across both physiological and cellular contexts. The in vivo data demonstrate the net outcome of this pathway under natural high-altitude conditions; the SMSC model allows us to dissect the underlying molecular events, including PINK1 and Parkin activation, initiation of autophagic flux, and compensatory remodeling of mitochondrial respiratory chain enzyme activities. Overall, these findings indicate that hypoxia is a critical factor in promoting autophagy in yak SMSCs and suggest that the enhanced autophagy observed in the skeletal muscle and lungs at higher altitudes is closely associated with hypoxia.

Alterations in the activities of key enzymes in the mitochondrial respiratory chain are essential indicators of mitochondrial impairment and energy metabolism. MRCCI is a core regulatory factor in mitochondrial oxidative phosphorylation [18] and is highly sensitive to fluctuations in oxygen concentration and ROS accumulation. CS, the rate-limiting enzyme of the tricarboxylic acid cycle, is a classical marker for assessing the total mitochondrial mass based on its enzymatic activity [49,50]. Our study demonstrated a consistent decrease in MRCCI and CS activity in hypoxia-exposed yak SMSCs, indicating a decrease in mitochondrial quantity in yak SMSCs under hypoxic conditions. Notably, there was a significant increase in SDH, COX-1, and CAT activities and PGC-1α expression under hypoxic conditions. Given that SDH is the first enzyme in the electron transport chain, changes in its activity directly indicate mitochondrial function [51]. The downregulation of MRCCI and CS reduces electron influx into the respiratory chain, thereby lowering mitochondrial membrane potential and electron leakage—a critical mechanism to limit excessive ROS production under hypoxia. COX-1 upregulation enhances the efficiency of oxygen utilization by cytochrome c oxidase [20]. The upregulation of SDH and COX-1 provides an alternative electron entry route and enhances oxygen utilization efficiency, as COX-1 has a higher affinity for oxygen and can sustain oxidative phosphorylation even under low oxygen tension. In addition, CAT antioxidant activity effectively lowers intracellular ROS levels, thereby promoting cell proliferation and migration [52]. As a key regulator of mitochondrial biogenesis, higher PGC-1α levels coordinate mitochondrial number, function, and biosynthesis [53]. Overall, these changes may reflect the adaptive response of yak SMSCs to hypoxia. This metabolic reprogramming allows yak SMSCs to sustain energy homeostasis and limit oxidative damage under hypoxic conditions. Such an adaptive trade-off represents an intensive adaptation strategy to high-altitude hypoxia. However, 3-MA-induced autophagy inhibition significantly downregulated SDH, COX-1, and CAT activities and PGC-1α levels, indicating that autophagy is essential for the removal of damaged mitochondria in yak SMSCs and acts as a key regulatory mechanism to maintain the homeostasis of critical enzymes in the mitochondrial respiratory chain.

Under hypoxic conditions, PINK1/Parkin-mediated mitophagy plays a vital role in preserving mitochondrial function and quality [54,55,56]. Previous studies have indicated that ROS accumulation induces Parkin expression [57,58]. PINK1 accumulation in damaged mitochondria triggers an autophagic program that eliminates abnormal mitochondria [59]. In the hypoxia model, we observed an increase in PINK1 and Parkin protein levels, initiation of autophagy, and a compensatory increase in the activity of key enzymes of the mitochondrial respiratory chain (SDH, COX-1, CAT, and PGC-1α). However, 3-MA-induced autophagy inhibition downregulated PINK1 and Parkin expression, upregulated p62 expression, inhibited autophagic flux, and suppressed the activity of key mitochondrial respiratory chain enzymes. Importantly, our study confirmed that PINK1/Parkin-mediated mitophagy is an important mechanism for maintaining mitochondrial function in yak SMSCs under hypoxic conditions. However, gene-specific knockout or overexpression techniques are necessary to confirm whether this pathway plays a pivotal role in hypoxic adaptation in yaks or is merely an epiphenomenon of the stress response.

## 5. Conclusions

Our study explored the association between mitophagy and high-altitude tolerance and mitochondrial metabolic enzyme activity in yaks using tissues from yaks at various altitudes and an acute hypoxic cell model. Importantly, PINK1/Parkin-mediated mitophagy plays a pivotal role in coordinating mitochondrial quality control and energy metabolic adaptation to hypoxia in yaks. Given that our current study primarily utilized pharmacological inhibition of autophagy, future investigations employing gene-specific knockout or overexpression of PINK1 and Parkin in yak SMSCs would provide definitive causal evidence for the role of this pathway in hypoxia adaptation. Overall, these findings establish PINK1/Parkin-dependent mitophagy as an intrinsic, cell-autonomous mechanism underlying high-altitude adaptation, provide insights into the molecular mechanisms of hypoxic adaptation in yaks, and serve as a reference for research on hypoxic adaptation in high-altitude human populations and other plateau animals.

## Data Availability

The original contributions presented in this study are included in the article. Further inquiries can be directed to the corresponding author.

## Supplementary Materials

The following supporting information can be downloaded at: https://www.mdpi.com/article/10.3390/ani16142121/s1. File S1. In vitro isolation, culture, and identification of skeletal muscle satellite cells from yak.

## Abbreviations

The following abbreviations are used in this manuscript:

CS	Citrate synthase
ROS	Reactive oxygen species
SMSCs	Skeletal muscle satellite cells
MRCC1	Mitochondrial respiratory chain complex I
SDH	Succinate dehydrogenase
COX-1	Cytochrome c oxidase subunit 1
TEM	Transmission electron microscopy
ELISA	Enzyme-linked immunosorbent assay
T-AOC	Total antioxidant capacity
CAT	Catalase
PGC1-α	proliferator-activated receptor-γ coactivator-1α
3-MA	3-methyladenine
