# Supplementary material for: PINK1/Parkin-Mediated Mitophagy Participates in High-Altitude Hypoxia Adaptation in Yaks via Energy Metabolism Remodeling
Source: Animals (Basel). 2026 Jul 8;16(14):2121. doi: 10.3390/ani16142121 (PMC13403501; doi:10.3390/ani16142121)
Supplement: Supplementary file 1 [file animals-16-02121-s001.zip › animals-4345371-supplementary.pdf]

Samples were obtained from a slaughterhouse in Guide County, Hainan Tibetan Autonomous Prefecture, Qinghai Province. A female yak grazing under natural conditions was slaughtered, and a fetus of approximately 4–5 months' gestation (without vital signs) was found in the uterus. Fetal leg skeletal muscle tissue was isolated with sterile surgical instruments. The sample was rinsed with 75% alcohol, then washed in 1× PBS containing 1% penicillin-streptomycin and finally stored in an ice box. It was transported to the laboratory within 2 h for cell isolation.

The experimental materials were obtained from by-products of conventionally slaughtered yaks. The animal experiment protocol was approved by the Animal Welfare and Research Ethics Committee of Qinghai University (number: SL-2023005).

Main Reagents and Instruments

Fetal Bovine Serum (FBS) (10099141C), product of Gibco, USA; Collagenase Type II (GC305014) and Penicillin-Streptomycin Solution (100×) (G4003), products of Wuhan Servicebio Technology Co., Ltd.; Epidermal Growth Factor (PF1101), product of Cellregen (Beijing) Life Science & Technology Co., Ltd.; 1× PBS Buffer (BL302A) and DMEM High Glucose Liquid Medium (BL304A), products of Beijing Lajieko Technology Co., Ltd.; 0.25% Trypsin Solution (HU-YM-100) , products of Guangzhou Hucheng Technology Co., Ltd.; Serum-Free Cell Freezing Medium (C40050), product of Suzhou Xinsaimei Biotechnology Co., Ltd.

The inverted fluorescence microscope was purchased from Nikon Corporation, Japan; the clean bench, CO_2 incubator, and microplate reader were purchased from Thermo Scientific, USA; the low-temperature ultracentrifuge was purchased from Eppendorf AG, Germany.

Reagent Preparation

Collagenase II Solution: Add 100 μL of sterile 1× PBS containing Ca2+ and Mg2+ to 100 mg of collagenase, vortex thoroughly until fully dissolved, and prepare a 1 g/mL (i.e., 1000×) storage solution. Then filter-sterilize using a low-protein-binding 0.22 μm filter membrane, aliquot into small portions, and store at -20°C in the dark. Before use, thaw on ice, avoid repeated freeze-thaw cycles, and use a 0.2% collagenase II solution to digest the cells.

DEME medium: Before use, add 1% penicillin-streptomycin mixture. To prepare 10% complete medium, add 5 mL of fetal bovine serum to every 45 mL of medium. The low-serum

medium contains 3% serum.

Isolation and Culture Steps of Skeletal Muscle Satellite Cells from Yak Fetal Bones

Take 0.5 cm^3 of the hind leg muscle tissue from fetal cattle, rinse three times with 1% double-antibody 1×PBS, cut the tissue into minced pieces using ophthalmic scissors, and repeatedly rinse with PBS. Let it stand for 1 minute, then aspirate the floating tissue. Centrifuge at 1500 r/min for 10 minutes and discard the supernatant. Add 2 mL of a mixture of 0.2% collagenase II and 0.25% trypsin, and digest in a 37° C water bath for 1 hour, mixing every 10 minutes.

After digestion is complete, centrifuge at 1500 rpm for 5 minutes, collect the precipitate, and discard the supernatant. Add 2 mL of 0.25% trypsin, resuspend, and place in a cell culture incubator for continued digestion. Mix every 5 minutes and observe the digestion process. Once the cells are dissociated into single, full cells, add complete culture medium (DMEM + 10% FBS + 1% antibiotics) to terminate digestion. Pass the cell suspension through a 200-mesh cell strainer, then a 400-mesh cell strainer. Centrifuge, discard the supernatant, add culture medium, and seed onto a cell culture dish. Incubate in a CO_2 cell culture incubator to obtain bovine skeletal muscle satellite cells (SMSCs).

Due to the different cell adhesion characteristics, the differential adhesion method can be used to purify yak skeletal muscle satellite cells. In the early stage, the cells are round with complete edges (Figure 1A). After 24 hours, the cells show clear proliferation, with a relatively high cell density (Figure 1B). When the first-generation cells reach an 80% confluence in the culture flask, they are passaged. The second-generation cells grow faster, and after 24 hours, the most common in vitro morphology of muscle satellite cells can be observed (Figure 1C): the myoblast stage. After the cells fully adhere, they are mainly spindle-shaped with clear edges. As the fusion rate increases, the cells tend to form vortex-like and parallel arrangements (Figure 1D). Over time in culture, short, thick multinucleated myotubes can be seen under the microscope. As the myotubes elongate and thicken further, they form long tubular or network structures (Figure 1E). With continuous passaging, the morphology of the fifth-generation muscle satellite cells changes (Figure 1F). The edges gradually become blurred and rounded, and some aging myotubes can contract and detach from

the bottom of the culture dish.

Cells in the first five passages maintained relatively stable morphology. At passage 6, morphological changes occurred in some culture flasks, with cells becoming elongated, spindle-shaped, or triangular and forming a net-like structure (Figure 1G); peripheral cells gradually deformed. After continued culture to passage 7, cells progressively differentiated into multiple morphologies (Figure 1H), appearing as large, flattened, round cells, some with irregular shapes. With prolonged culture, cell morphology became increasingly blurred, edges swelled, and the capacity for myotube differentiation diminished. All cells utilized in this study were confined to the initial five passages.

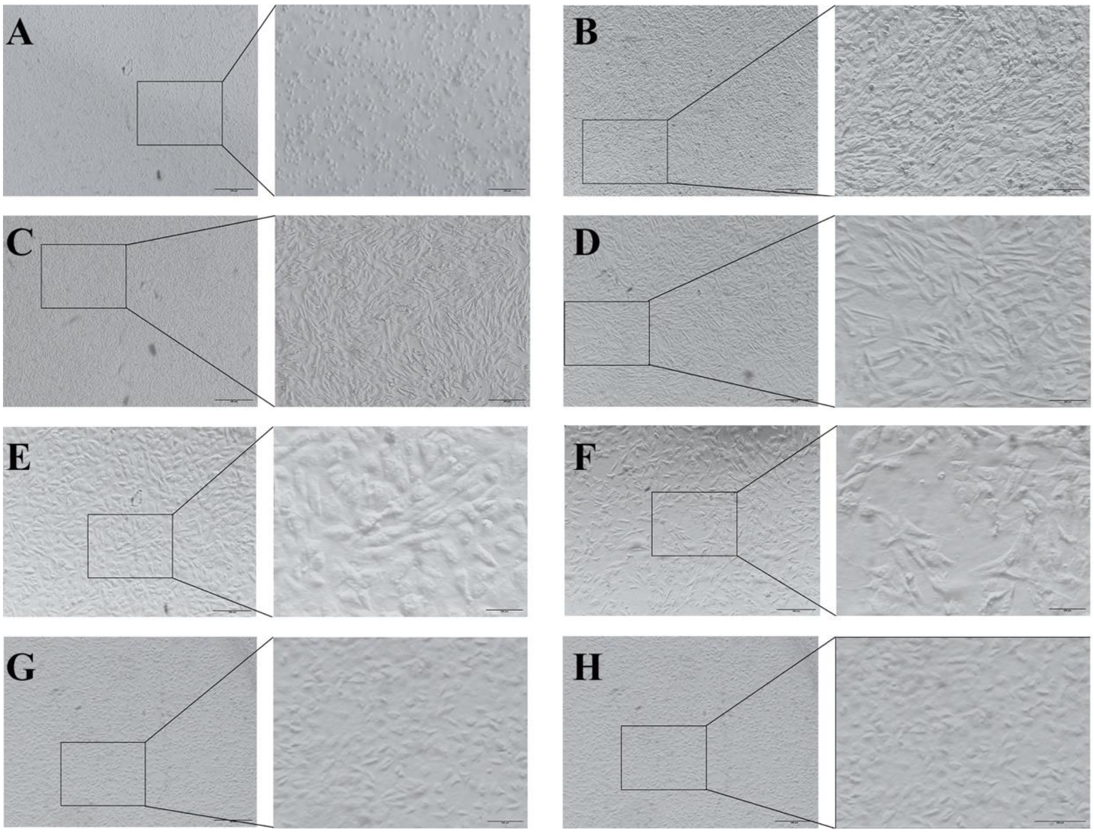

A: Cells extracted at 0 hours; B: Cells cultured for 24 hours; C: Second-generation cell culture; D: Third-generation cell culture; E: Fourth-generation cell culture; F: Fifth-generation cell culture; G: Sixth-generation cell culture; H: Seventh-generation cell culture

Figure S1 Microscopic Observation of Skeletal Muscle Satellite Cells in Yaks（10×）
